# Is the Sexual Behaviour of HIV Patients on Antiretroviral therapy safe or risky in Sub-Saharan Africa? Meta-Analysis and Meta-Regression

**DOI:** 10.1186/1742-6405-9-14

**Published:** 2012-05-14

**Authors:** Asres Berhan, Yifru Berhan

**Affiliations:** 1 Hawassa University, Hawassa, Ethiopia; 2 Hawassa University College of Medicine and Health Sciences, P.O. Box 1560, Hawassa, Ethiopia

**Keywords:** Antiretroviral therapy, Meta-analysis, Meta-regression, Multiple sexual partners, Unprotected sex, Sub-Saharan Africa

## Abstract

**Background:**

Reports on the sexual behavior of people on antiretroviral therapy (ART) are inconsistent. We selected 14 articles that compared the sexual behavior of people with and without ART for this analysis.

**Methods:**

We included both cross-sectional studies that compared different ART-naïve and ART-experienced participants and longitudinal studies examining the behavior of the same individuals pre- and post-ART start. Meta-analyses were performed both stratified by type of study and combined. Outcome variables assessed for association with ART experience were any sexual activity, unprotected sex and having multiple sexual partners. Random-effect models were applied to determine the overall odds ratios. Sub-group analyses and meta-regression analyses were performed to examine sources of heterogeneity among the studies. Sensitivity analysis was also conducted to evaluate the stability of the overall odds ratio in the presence of outliers.

**Results:**

The meta-analysis failed to show a statistically significant association of any sexual activity with ART experience. It did, however, show an overall statistically significant reduction of any unprotected sex, having multiple sexual partners and unprotected sex with HIV negative or unknown HIV status with ART experience. Meta-regression showed no interaction between duration of ART use or recall period of sexual behavior with the sexual activity variables. However, there was an association between the percentage of married or cohabiting participants included in a study and reductions in the practice of unprotected sex with ART.

**Conclusion:**

In general, this meta-analysis demonstrated a significant reduction in risky sexual behavior among people on ART in sub-Saharan Africa. Future studies should investigate the reproducibility and continuity of the observed positive behavioural changes as the duration of ART lasts a decade or more.

## Introduction

Across the globe, the introduction of antiretroviral therapy (ART) has saved millions of life since it was widely introduced in developed countries in 1996 and in sub-Saharan Africa in 2002 [1]. ART contributed to a 19% decline in deaths from HIV infection from 2004 to 2009 [1]. Sub-Saharan region bears 68% of the global HIV burden. By 2009, about 37% of HIV-infected persons in sub-Saharan countries who were eligible for ART were able to access antiretroviral drugs. Just as in developed countries, ART has improved quality of life and survival in sub-Saharan Africa [2-4].

As severely ill HIV patients recover on ART, they feel general wellbeing and their sexual desire is likely to resume. Is the sexual behavior of HIV patients on ART safe or risky? Reports on sexual behaviors of people on ART have been inconsistent; six studies African and non-African countries have shown that patients on ART are more likely to engage in unprotected sex [5-10]; however, other cross sectional and longitudinal studies have reported a decrease in rates of risky sexual behavior after ART [11-15].

In a systematic review of three articles, Kennedy et al found a significant reduction in sexual risk-taking behaviour among patients on ART in developing countries [16]. One very recent cross-sectional study from Ethiopia showed that more than one third (36.9%) of study participants reported to have unprotected sexual intercourse in three months period prior to data collection [17]. Another cross-sectional study among patients on ART from an informal urban settlement in Kenya found increased rates of inconsistent condom use among women and increased rates of multiple sexual partners among married men [18]. A 2004 meta-analysis of studies from developed countries found that rates of unprotected sex among people on ART were not significantly different from those among HIV-infected people not on ART [19]. It found high rates of unprotected sex, however, among those who believe that they will not transmit HIV while on ART [19].

No previous meta-analysis has been published on the sexual behaviors of people on ART in Sub-Saharan Africa. This meta-analysis and meta-regression was conducted based on studies comparing the sexual behavior of people on ART with HIV-infected people not on ART. We hope this analysis sheds more light on the gradient of risky sexual behaviours among people on ART in Sub-Saharan Africa.

## Methods

### Search strategy

We conducted a computer search of PubMed, Google Scholar, WHO (HINARI) for articles on risky sexual behavior in relation to ART in sub-Saharan Africa published from January 2000 to October 2011. We obtained article texts either free on-line or from HINARI [20]. Our search terms included “HAART”, “Highly active antiretroviral therapy”, “ART”, “Antiretroviral therapy”, “sexual activity”, “unprotected sex”, “multiple sexual partners and unprotected sex with HIV negative or unknown HIV status”, “inconsistent condom use,” and the names of Sub-Saharan African countries. We also searched the reference lists of articles identified through the computer search.

### Article selection

Articles that fulfilled the following three criteria were included in our meta-analysis: must be published in English, must be conducted in sub-Saharan Africa, and must compare either the sexual behavior of HIV-positive people on ART with that of HIV-positive, ART naïve or the sexual behavior of HIV-positive individuals before ART initiation with the sexual behavior of the same individuals after starting ART.

Our search criteria initially identified 53 complete journal articles on risky sexual behavior of people on ART in sub-Saharan Africa. Based on our inclusion criteria, only 14 of these articles qualified for the meta-analyses. The major reasons for the exclusion of 39 articles were: lack of comparative data on sexual behavior with behavior prior to starting ART or with an ART-naïve people control group; focused solely on knowledge and attitudes without examination of behavior, and review articles that included no data collection. Of the 14 selected articles, six were cross-sectional studies comparing the sexual behaviour of ART naïve with ART experienced people and eight were prospective cohort studies comparing behavior of the same individuals before and after starting ART. General information on the studies included is shown in Table 1.

**Table 1:** General information on the studies included, 2011

**Reference**	**Country**	**Type of study**	**Type of comparison**	**Sampled**
Wandera B et al. [21]	Uganda	Prospective	Pre-ART vs Post ART	559 ART-naïve
Luchters S et al. [22]	Kenya	Prospective	Pre-ART vs Post ART	234 ART-naïve
Moatti J P et al. [23]	Coˆte d’Ivoire	Cross-sectional	ART experienced vs. ART naïve	547 ART naïve and 164 ART experienced
Bateganya M et al. [24]	Uganda	Cross-sectional	ART experienced vs. ART naïve	354 ART naïve and 369ART experienced
Pearson C R et al. [25]	Mozambique	Prospective	Pre-ART vs Post ART	277 ART naive
Bunnell R et al. [26]	Uganda	Prospective	Pre-ART vs Post ART	926 ART-naive
Venkatesh K K et al study 1, [27]	South Africa	Prospective	Pre-ART vs Post ART	6263 ART-naive
Eisele T P et al. [28]	South Africa	Cross sectional	ART experienced vs. ART naïve	404 ART naïve and 520 ART experienced
Dia A et al. [29]	Cameroon	Cross-sectional	ART experienced vs. ART naïve	216 ART naïve and 691 ART experienced
Peltzer K et al. [30]	South Africa	Prospective	Pre-ART vs Post ART	735 ART naïve
Sarna A et al. [31]	Kenya	Cross-sectional	ART experienced vs. ART naïve	143 ART naïve and 179 ART experienced
Venkatesh K K et al study2 [32]	South Africa	Cross-sectional	ART experienced vs. ART naïve	247ART-naïve and 1295 ART experienced
Diabate S et al [8]	Coˆte d’Ivoire	Prospective	Pre-ART vs Post ART	312 ART naïve
McClelland R. S et al. [33]	Kenya	Prospective	Pre-ART vs Post ART	898 ART naïve

### Data extraction

From the studies that met the inclusion criteria, the following data were abstracted: year of publication, country where conducted, setting, type of comparison [within individuals (Pre- ART vs. Post- ART) or between individuals (ART naïve vs. ART experienced)], sample size, sexual history (sexually activity, multiple sexual partners, unprotected sex with HIV positive/HIV negative or unknown HIV status, and inconsistent condom use), recall period for sexual activities, duration on ART and proportion of married participants.

### Operational definition

In this article, we use the terms “ART-naïve” and “ART-experienced” only with respect to studies comparing behavior among different individuals. We use the terms “pre-ART” and “post-ART” only with respect to studies comparing behavior within the same individuals. We did not attempt to standardize definitions of these terms; we used them as used in each of the articles selected. We use the term “unprotected sex” to mean either sexual activity without the use of a condom or inconsistent condom use with any partner irrespective of HIV serostatus. We use the term “multiple sexual partners” to mean sexual activity with two or more sex partners during the study period. In this analysis, all the study participants were HIV positive. All articles selected for this meta-analysis have got ethical clearance from their respective institution.

### Statistical analysis

We used Meta Analyst (Beta 3.13) software [34] and Comprehensive Meta Analysis software [35] to carry out meta-analysis and meta-regression analysis, respectively. We conducted four meta-analyses: on the association of ART experience with any sexual activity, on the association of ART experience with any unprotected sex, on the association of ART experience with having multiple sexual partners and on the association of ART experience with having unprotected sex with partners who were HIV negative or of unknown HIV status. We used the DerSimonian-Laird method (Random-effect model) to calculate the overall odds ratios. To determine the heterogeneity among the studies, it was possible to generate values for Tau^2^, Chi-square (Q), I^2^ and P-value. For this analysis, when the value of I^2^ exceeded 50%, we considered the total variation across the studies to be large and to be due to heterogeneity rather than to chance [36].

In the presence of significant heterogeneity, summary estimates from meta-analyses are believed to be difficult to interpret; and Lijmer et al recommends investigating heterogeneity using subgroup (stratified) analysis and meta-regression analysis [37] Therefore, to examine sources of heterogeneity among the studies, we performed sub-group analyses with Meta Analyst (Beta 3.13) software and meta-regression analysis with Comprehensive Meta Analysis software. Sub-group analysis was carried out at two stages: first, stratifying by type of study (among individuals vs. within individuals); second, combining all studies of both types. Random effects linear meta-regression analysis was performed with three covariates or moderators: duration on ART, recall period for sexual activities and percentage of married participants in the group receiving ART.

Sensitivity analysis was also conducted to see the stability of the overall odds ratio to inclusion of individual studies. For all statistical tests, P-value ≤ 0.05 was considered as statistically significant.

## Results

**Figure 1:**
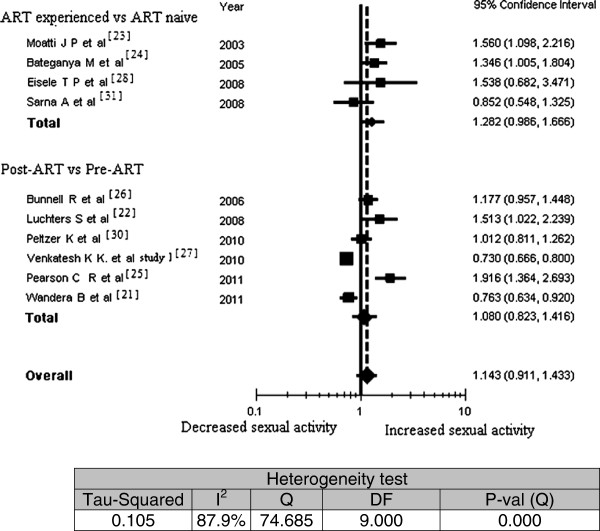
Meta-analysis of the association between antiretroviral therapy experience and any sexual activity; Pooled effect estimate from a random-effects model, 2011.

The studies making comparisons among individuals included 5129 participants (1911 ART-naïve participants and 3218 ART–experienced participants). The studies making within-individual comparisons included 1024 participants were included. The participants’ duration on ART ranged from 3–36 months (median six months and interquartile range 6–12 months). Figure 1 presents the results of the meta-analysis of any sexual activity in relation to ART experience. Although two studies [23,24] showed a statistically significant increase in sexual activity among ART-experienced as compared with ART-naïve participants, the overall analysis portrayed no association of sexual activity in either of the study types (OR = 1.14; 95% CI, 0.91 to 1.43). However, the proportion of sexually active people was higher among ART-experienced than ART-naïve participants in three of the four studies that made comparisons among individuals [23,24,28]. 

The within-individual studies were more inconsistent than studies that made comparisons between individuals. Two studies [21,27] found statistically significant decreases in sexual activity post-ART while another two studies [22,25] found increased sexual activity pre-ART.

Sensitivity analysis attested to the stability of the overall odds ratio; it oscillated in the range of 1.07 to 1.21 (changed by ± 0.07 from the overall odds ratio). The test of heterogeneity showed a significant variation among the included studies (Q = 74.7; I^2^ = 87.9%), signifying that the variation was due to heterogeneity rather than chance. The impact of duration on ART and recall time on the overall odds ratio was assessed by the random effect meta-regression model. As shown in Table 2, these variables were not found to have a significant effect; the regression coefficient of the log odds ratio of sexual activity was not significantly different from zero in either the analysis of average time while on ART (regression coefficient = −0.016; 95% CI, -0.033 to 0.002; P = 0.078) or the analysis of recall time (regression coefficient = 0.042; 95% CI, -0.020 to 0.104; P = 0.185).

**Table 2:** Summary of meta-regression analysis findings, 2011

**Variable**	**Regression coefficient**	**Standard error**	**95% CI**	**P-value**
Effect of duration on ART on sexual activity	- 0.016	0.009	−0.033, 0.002	0.078
Effect of time of recall on sexual activity	0.042	0.032	−0.020, 0.104	0.185
Effect of time of recall on unprotected sex	0.0029	0.078	−0.151, 0.157	0.97
Effect of duration on ART on unprotected sex	−0.0328	0.018	0.069, 0.003	0.076
Effect of married participants on Unprotected sex	−0.032	0.014	−0.059, -0.004	0.023
Effect of time of recall on multiple sexual partner	0.018	0.047	−0.074, 0.111	0.697
Effect of duration on ART on multiple sexual partners	0.0457	0.037	−0.028, 0.119	0.22
Effect of married participants on multiple sexual partners	0.0313	0.0249	−0.017, 0.080	0.21

**Figure 2:**
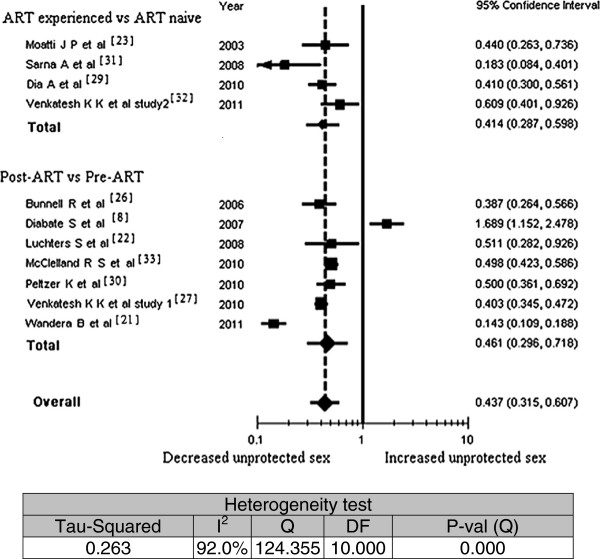
Meta-analysis of the association between antiretroviral therapy experience and any unprotected sex; Pooled effect estimate from a random-effects model, 2011

As shown in Figure 2, all studies [21-23,26,27,29-33] but one showed a statistically significant reduction in rates of unprotected sex among people on ART. The divergent study showed a statistically significant increase in unprotected sex among those on ART. The overall odds ratio in the meta-analysis demonstrated a statistically significant reduction in unprotected sex while on ART (OR = 0.437; 95% CI, 0.315 - 0.607). The association appears a bit stronger in the studies comparing rates of unprotected sex among different individuals (OR = 0.414; 95% CI, 0.287 - 0.598). In the sensitivity analysis, the overall odds ratio was very little changed by omitting individual studies (maximum ± 0.06). 

The studies included in the meta-analysis of unprotected sex also found to have significant variation due to heterogeneity rather than to chance (Q = 124.4; I^2^ = 92.0%). Meta-regression found no statistically significant interaction between unprotected sex and duration on ART (regression coefficient = −0.0328; 95% CI, -0.069 to 0.003; P = 0.076) or between unprotected sex and recall time (regression coefficient = 0.0029; 95% CI, -0.151 to 0.157; P = 0.97). But the meta-regression incorporating six studies [21,22,26,27,30,31] which analyzed the relationship of rates of unprotected sex with the percentage of married or cohabiting participants included in the study demonstrated a significant reduction of unprotected sex as the percentage of married participants increases (regression coefficient = −0.032; 95% CI,-0.059 to −0.004; P = 0.023).

**Figure 3:**
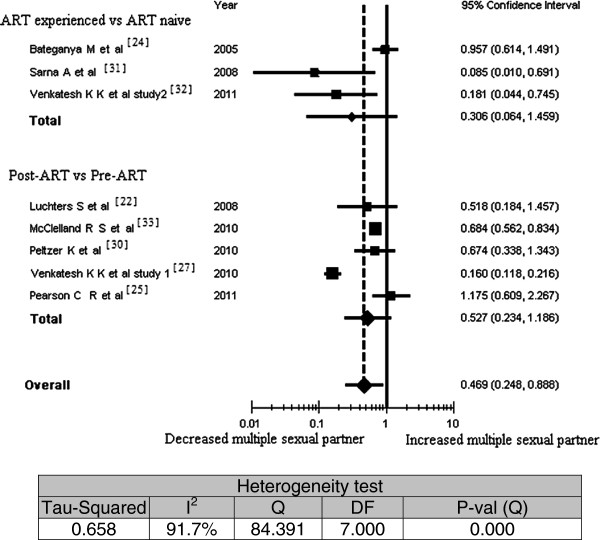
Meta-analysis of the association between antiretroviral therapy experience and having multiple sexual partners; Pooled effect estimate from a random-effects model, 2011.

Figure 3 shows the results of the meta-analysis of the relationship of ART experience to rates of multiple sexual partners. The overall odds ratio, including all eight studies which reported this information, indicated that rates of multiple sexual partners were significantly decreased among people on ART (OR = 0.47; 95% CI, 0.248 to 0.888). When considered separately by study type, however, the combined odds ratios were not statistically significant, although two studies of each type [27,31-33] independently showed a statistically significant reduction in rates of multiple sexual partners with ART experience. In other words, rates of multiple sexual partners were not found to be significantly different either when comparing ART-experienced people to ART-naïve people; or when comparing the same individuals’ behavior before and after ART initiation. The sensitivity analysis supports the findings of the sub-group analysis; exclusion of Sarna and *et al.* study from the analysis leads to a finding of an insignificant association of ART experience with rates of having multiple sexual partners. 

Linear meta-regression analysis showed that neither duration of ART (regression coefficient = 0.0457; 95% CI, -0.028 to 0.119; P = 0.22), recall time (regression coefficient = 0.018; 95% CI, -0.074 to 0.111; P = 0.697) nor percentage of married participants (regression coefficient = 0.0313; 95%CI, -0.017 to 0.080; P = 0.21) were significantly associated with having multiple sexual partners.

**Figure 4:**
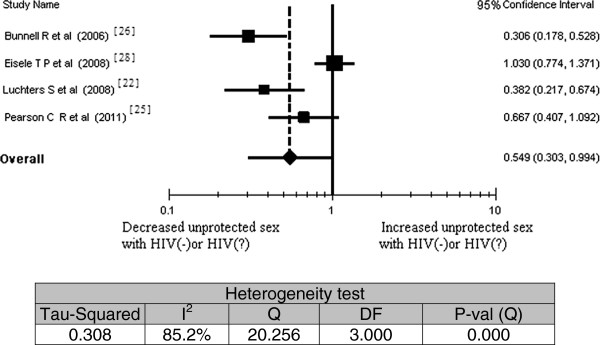
Meta-analysis of the association between antiretroviral therapy (ART) experience and unprotected sex with HIV negative or unknown HIV status partners; Pooled effect estimate from a random-effects model, 2011

Of the 14 studies included, only four [22,25,26,28] reported rates of unprotected sex with HIV-negative or unknown HIV status persons (Figure 4). The meta-analysis of the relationship between ART experience with rates of unprotected sex with HIV negative or unknown HIV serostatus showed a statistically significant reduction among people on ART (OR = 0.55; 95% CI, 0.303 to 0.994). 

## Discussion

Because of several contradictory reports, the issue of sexual behaviour of people on ART in sub-Saharan Africa still continues to be debatable. This meta-analysis is the first to provide a pooled quantitative estimate of the sexual behavior of people on ART by categorizing and analyzing the studies by type. In other words, the strength of this analysis is that it examined the sexual behavioral change with ART within individuals (pre-ART vs. post-ART study type) and among different individuals (ART-naïve vs. ART-experienced study type), and finally, combining the two study types (HIV-infected people not on ART vs. HIV-infected people on ART). We would expect the within-individual studies to be less affected by confounding variables due to variations between the individuals included than the studies that compared different ART-naïve and ART-experienced individuals. Our analysis also permitted exploration of different dimensions of the sexual behavior of HIV infected patients. Although we found no statistically significant difference in sexual activity among HIV-infected people on ART, six of the 10 studies [22-24,26-28] which reported this variable reported an increase in sexual activity, which is consistent with other report [38]. However, the statistically significant reduction of unprotected sex among those on ART found in both prospective and cross sectional studies is inconsistent with the results of the meta-analysis of studies in developed nations, which found no statistically significant difference in this behavior [19]. Furthermore, this analysis also revealed the reduction in rates of multiple sexual partners and rates of unprotected sex with HIV-negative or unknown HIV status partners by people on ART. This finding may trigger questions as to why risky sexual behavior decreases with ART in sub-Saharan Africa while remaining unchanged in developed nations. One reason may be that risky sexual practices in sub-Saharan Africa were extremely prevalent at the outset, which we know because sub-Saharan Africa is the region most affected by HIV and because the majority of HIV infections in this region are attributed to unprotected heterosexual intercourse [2,39].

In addition, one may argue that the dramatic improvement in their health after beginning ART may have given HIV patients the hope of living long. Their previous sufferings had been an extremely bad experience; and professionals probably counseled them not to put themselves and their partners at risk of infection with new HIV strains, with HIV strains resistant to antiretroviral drugs and with other sexually transmitted infections. In support of this hypothesis, a qualitative study from Uganda reported that many ART patients were worried about risking their improving health through HIV super-infection and about the risk of resuming sexual practice [40]. The positive behavioral change among ART patients in Sub-Saharan Africa may also be the effect of good adherence to clinic visits, which will provide the chance to refresh their knowledge on HIV and to receive further warnings during every visit. A study conducted in six public HIV clinics has established the association of good ART adherence with decreased risky sexual behaviors [41].

A meta-analysis on adherence to ART in Sub-Saharan Africa and North America found better adherence among Sub-Saharan Africans [42]. The observed good adherence in Sub-Saharan Africa may have contributed as well to avoidance of risky sexual practice. The reported positive behavioral change towards safer sex not only provides HIV patients an advantage in improving the quality of their lives but also reduces their chance of transmitting HIV to their partners and of acquiring new and resistant HIV strains.

There are some studies from Sub-Saharan Africa which report the association of higher rates of unprotected sex with shorter duration of ART therapy (the longer people stay on ART, the lower their rates of unprotected sex) [18,38,43]. However, our meta-regression analysis showed no statistically significant interaction of rates of unprotected sex with duration on ART. This finding also does not support the idea proposed by Womoyi et al, that patients who have just started ART may experience illness which could decrease sexual desire and that as time passes and symptoms of HIV disease are resolved, risky sexual practice could emerge [40].

Our meta-regression also found no significant relationship between sexual activity and the time period over which participants were asked to recall such activity. However, the meta-regression of rates of unprotected sex in relation with the percentage of participants who were married found a significant interaction. The higher the percentage of married participants in a study, the lower the rates of unprotected sex found. This finding would be expected and may explain why the majority of studies included in this analysis found a reduction in unprotected sex, but other reports have not [18].

One limitation of our analysis is that all the primary studies analyzed used interviews to collect data on sexual behavior; this is open to recall bias and social desirability bias [44]. First, in some settings, openly discussing sexual issues may be taboo or interviewees may fear losing value in society. Second, patients on ART, many of whom may have been thoroughly counseled about the consequences of unprotected sex at ART initiation and in subsequent visits, may not feel comfortable reporting truthfully about their subsequent sexual behavior. Another limitation is the lack of data provided by the primary studies. Most of the primary studies failed to include data on stages of HIV disease (such as viral load, CD4 count, clinical condition) and level of medication adherence. Therefore we were unable to analyze the effect of these parameters on sexual behavior of people with and without ART. Many of the primary studies also lacked information on whether married participants had more than one sexual partner. The variation in percentage of married participants in the primary studies does not seem adequate, on its own, to explain variations among the findings. Other sources of heterogeneity, which we did not analyze, could be average age, education level, sex allocation, income, CD4 count and culture of the participants.

In conclusion, the significant reduction in risk taking sexual behavior among people on ART regardless of duration on ART is consistent with several individual studies and a systematic review from developing countries [16,45]. However, it was not possible to identify what contributes to this positive behavioral change, and we are not sure whether the observed behavioral change is sustainable and representative of all sub-Saharan African countries. Recently published non-comparative studies from Sub-Saharan African countries reported that many HIV patients on ART continue to exhibit high risk sexual behavior [10,17,18,46]. As a non-comparative cohort study of people on ART from Uganda showed, risky sexual behaviours were declining before 2004 but thereafter were on the upswing [47]. Although our meta-analysis from comparative primary studies does support some previous research, it is probably premature to conclude that there has been region-wide significant reduction in unprotected sex among people on ART in Sub-Saharan Africa, which were also a concern of other authors [26]. Furthermore, it is known that regardless of ART, men and women have different patterns of sexual activity. However, in this meta-analysis, it was not possible to identify the sexual behaviour differences by men and women; out of eight studies that reported sexual activity, the data set in four was unfit for doing meta-analysis. Therefore, future studies should address the reproducibility of the observed positive behavioral changes in other settings and assess whether they continue as patients stay on ART for a decade or more.

## Competing interest

Authors would like to declare that there was no competing interest associated with this manuscript.

## Authors’ contributions

Both authors (AB and YB) actively involved in designing, articles search, articles selection, and manuscript writing. The contribution of AB was immense in doing the meta-analysis and meta-regression. Both authors read and approved the final manuscript.
